# The Effect of Dry Eye Disease on Scar Formation in Rabbit Glaucoma Filtration Surgery

**DOI:** 10.3390/ijms18061150

**Published:** 2017-05-28

**Authors:** Hong Ji, Yingting Zhu, Yingying Zhang, Yu Jia, Yiqing Li, Jian Ge, Yehong Zhuo

**Affiliations:** State Key Laboratory of Ophthalmology, Zhongshan Ophthalmic Center, Sun Yat-sen University, Guangzhou 510060, China; jihong@gzzoc.com (H.J.); zhuyingting@gzzoc.com (Y.Z.); zhangyingying@gzzoc.com (Y.Z.); jiayu@gzzoc.com (Y.J.); yqlee95@outlook.com (Y.L.); gejian@mail.sysu.edu.cn (J.G.)

**Keywords:** dry eye disease, scar formation, glaucoma, filtration surgery, rabbit

## Abstract

The success rate of glaucoma filtration surgery is closely related to conjunctival inflammation, and the main mechanism of dry eye disease (DED) is inflammation. The aim of this study was to evaluate the effect of DED on bleb scar formation after rabbit glaucoma filtration surgery. Sixteen New Zealand white rabbits were randomly divided into control and DED groups. A DED model was induced by twice-daily topical administration of 0.1% benzalkonium chloride (BAC) drops for three weeks. Ocular examinations were performed to verify the DED model. Surgical effects were assessed, and histologic assessments were performed on the 28th postoperative day. Higher fluorescein staining scores, lower basal tear secretion levels and goblet cell counts, and increased interleukin 1β (IL-1β) levels were observed in the DED group. The DED eyes displayed significantly higher intraocular pressure (IOP)% on the 14th postoperative day; a smaller bleb area on days 14, 21 and 28; and a shorter bleb survival time. Moreover, proliferating cell nuclear antigen (PCNA) and alpha-smooth muscle actin (α-SMA) levels were significantly increased in the DED group. These results demonstrate that DED promotes filtering bleb scar formation and shortens bleb survival time; these effects may be mediated via IL-1β.

## 1. Introduction

Glaucoma is a major eye disease that results in blindness [1] whose first-line surgical treatment is filtration surgery. Due to increases in fibroblast activity, which impairs the formation of filtering blebs and reduces aqueous humor outflow, the failure rate of this surgery can reach 15–30% [2,3]. Conjunctival inflammation is an important contributor to the development of fibrosis. When corneal and conjunctival tissues are exposed to an inflammatory milieu, fibroblasts will undergo transformation into myofibroblasts, which leads to increases in proliferation and tissue remodeling [4]. In 2007, the Dry Eye Work Shop (DEWS) updated the definition of dry eye disease (DED).The new definition emphasized that DED must be accompanied by ocular surface inflammation [5]. Glaucoma patients are at risk for developing DED. Baudouin et al. [6] reported that the prevalence rates of severe ocular surface disease in patients with severe and mild glaucoma were 63% and 41%, respectively. The reasons include that glaucoma typically affects elderly individuals, and intraocular pressure (IOP)-lowering drugs with preservatives are widely used in patients with glaucoma.

The success rate of filtration surgery is closely related to the severity of conjunctival inflammation and scarring. Bleb failure occurs more easily when fibroblasts and inflammatory cell levels increase [7]. It has been reported that treatment with topical nonsteroidal anti-inflammatory drugs (NSAIDs) or steroids before trabeculectomy, which suppress conjunctival inflammation, may improve surgical outcomes [8]. The core mechanism underlying the development of DED is inflammation [9]; thus, DED may be related to the results of filtration surgery. A previous study [10] showed that failed glaucoma surgeries featured lower MUC5AC levels (MUC5AC, a goblet cell-derived gel-forming mucin, plays a vital role in maintaining tear film stability [11]) and higher HLA-DR levels (HLA-DR, the most abundant inflammatory biomarker on the ocular surface, is overexpressed in DED and may thus serve as a method for classifying DED severity and evaluating treatment responsiveness [12]) than successful surgeries. The expression levels of the above proteins may therefore become predictors of filtering surgery outcomes. However, the effect of DED on bleb scar formation in glaucoma filtration surgery has not been well studied. In the current study, we investigated whether DED promotes bleb fibrosis in rabbit eyes.

## 2. Results

### 2.1. Inflammatory Index

**Figure 1:**
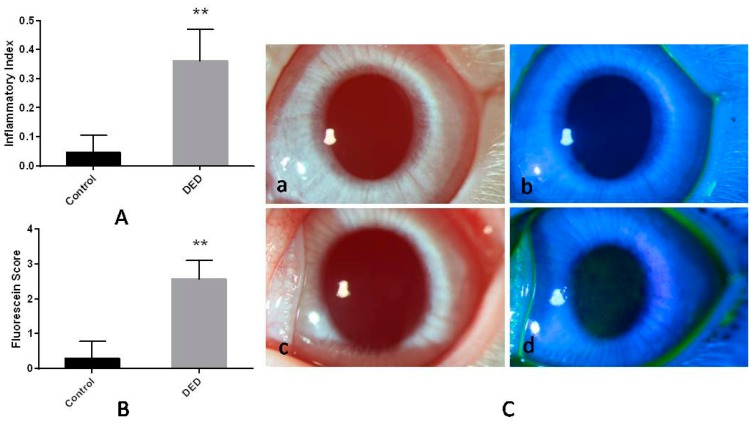
In vivo observations of ocular surface inflammation and corneal fluorescein staining in control and dry eye disease (DED) eyes. (**A**) The inflammatory index in the two groups. The DED group had a significantly higher inflammatory index than the control group; (**B**) the corneal fluorescein staining scores in the two groups. The DED group had a significantly higher fluorescein staining score than the control group; (**C**) photographs of corneas stained with and without fluorescein sodium. Signs of ocular abnormalities were not observed in the control eyes (**a**,**b**). Corneal edema and diffuse epithelial disruption were observed in the DED eyes (**c**,**d**) (16×). ** *p* < 0.01.

Symptoms (redness and photophobia) and signs (conjunctival congestion, mild-to-moderate corneal edema, and corneal epithelial damage) of ocular irritation were clearly present in the DED group after 0.1% benzalkonium chloride (BAC) treatment, and there was a significant difference in the inflammatory index between the DED and control groups (*p* <0.01, Figure 1A).

### 2.2. Corneal Fluorescein Staining

Corneal edema was observed in the DED group after 0.1% BAC treatment. Fluorescein staining revealed the presence of extensive epithelial disruption in the DED group, and DED eyes displayed significantly higher fluorescein staining scores than control eyes (*p* < 0.01, Figure 1B,C).

### 2.3. Schirmer’s Tear Test

**Figure 2:**
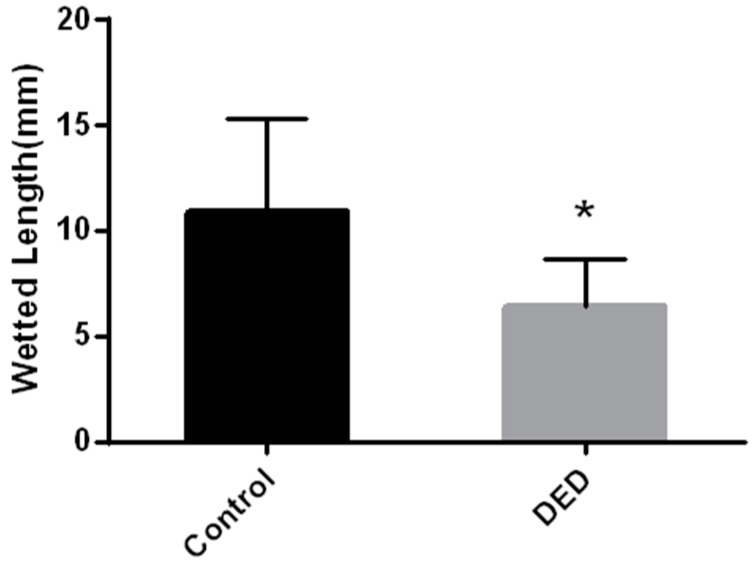
Wetted lengths, as determined by Schirmer’s tear tests, in the two groups. Basal tear secretion levels were significantly decreased in DED eyes compared with those in control eyes. * *p* < 0.05.

Schirmer I tests were subsequently performed in the control and DED groups. Basal tear secretion levels were significantly decreased in 0.1%BAC-treated eyes compared with levels in untreated eyes (*p* < 0.05, Figure 2).

### 2.4. Conjunctival Impression Cytology (CIC) and Goblet Cell Counts

**Figure 3:**
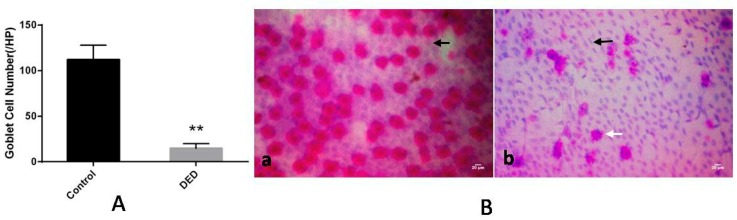
Conjunctival impression cytology (CIC) results for the eyes in the control and DED groups. (**A**) Goblet cell counts in the two groups. Compared with the control group, goblet cell counts were significantly decreased in the DED group; (**B**) CIC images for the two groups. The CIC grade was 0 in the control eyes (**a**), which featured normal-shaped conjunctival epithelial cells, large and round nuclei (black arrow), and plump goblet cells. The CIC grade was 2 in the DED eyes (**b**), which displayed squamous epithelial metaplasia, polygonal epithelial cells with smaller nuclei (black arrow), significantly decreased numbers of normal goblet cells, and irregularly shaped goblet cells (white arrow) (400×). ** *p* < 0.01. Scale bar = 20 μm.

Goblet cell counts were significantly lower in the DED group than in the control group after 0.1%BAC treatment (*p* < 0.01, Figure 3A). According to the Nelson system [13], the eyes in the control group displayed normal-shaped conjunctival epithelial cells, large and round nuclei, and plump goblet cells. Thus, the CIC grade for these eyes was 0. In contrast, squamous epithelial metaplasia, polygonal epithelial cells with smaller nuclei, significantly decreased numbers of normal goblet cells, and irregular goblet cells were noted in the DED group. Thus, the CIC grade for these eyes was 2 (Figure 3B).

### 2.5. Immunofluorescence for IL-1β

**Figure 4:**
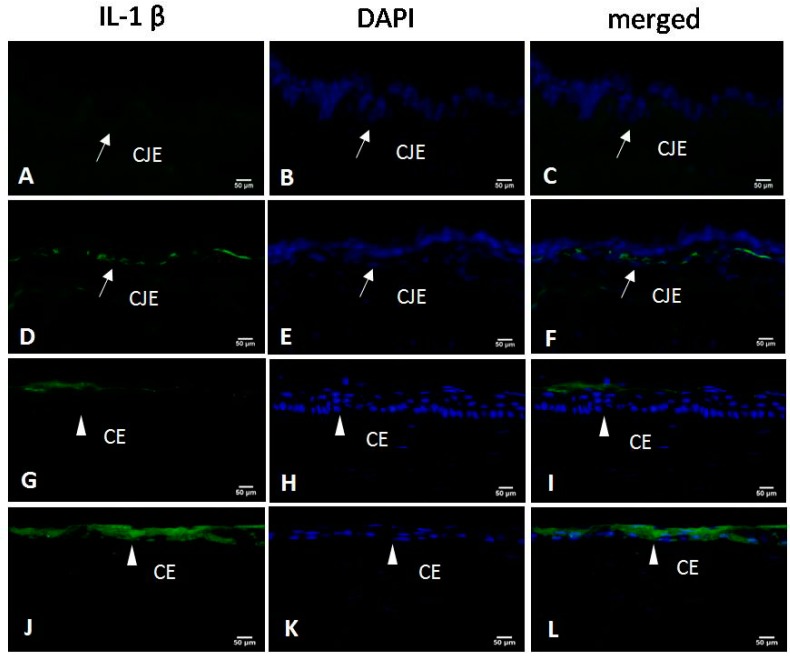
Immunofluorescence staining with interleukin-1β (IL-1β)-specific antibodies in the conjunctival epithelium (CJE) and corneal epithelium (CE) (IL-1β staining: green; nuclear staining: blue). In the control group, IL-1β staining was very weak in the CJE (**A**–**C**) and localized only to the surface of the CE (**G**–**I**). CJE and CE were thinner in the DED group, in which the basal layer of the CJE (**D**–**F**) and the entire CE (**J**–**L**) stained positive for IL-1β (×400). CJE: conjunctival epithelium (white arrow); CE: corneal epithelium (white triangle). Scale bar = 50 μm.

Higher interleukin 1β (IL-1β) levels were noted in the conjunctiva and cornea of the DED eye. In the DED eye, the conjunctival and corneal epithelia became thinner. IL-1β staining was localized to the basal layer of the conjunctival epithelium (CJE) and the entire corneal epithelium (CE). However, IL-1β staining was localized only to the surface of the CE and was very weak throughout all of the layers of the CJE in the control eye (Figure 4).

### 2.6. Clinical Evaluations after Glaucoma Filtration Surgery

The postoperative wound healed well in the two groups, and complications such as endophthalmitis, bleb leakage or cataracts were not observed. Conjunctival congestion was observed in all of the rabbits and resolved on day 21 or 28 after surgery. Mild-to-moderate anterior chamber flare was observed in all of the rabbits on the 1st postoperative day and gradually resolved within five days after surgery. Hyphema was observed in one case in the control group and two cases in the DED group on the first day. Blood located near the iris incision and peripheral iridectomy was the main reason for hyphema. Corneal edema was noted in two cases in the DED group and resolved within seven days after surgery. 

### 2.7. Intraocular Pressure

**Figure 5:**
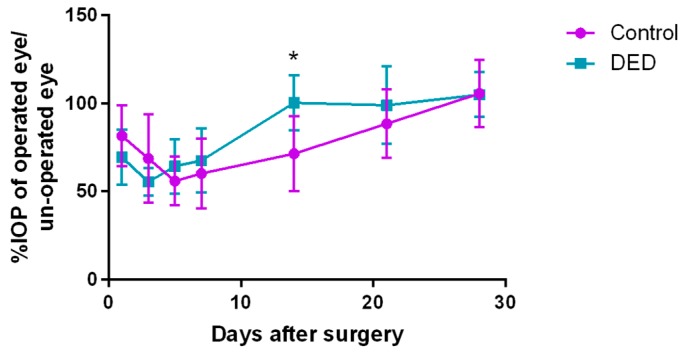
Postoperative intraocular pressure (IOP)% changes over 28 days in the two groups. The IOP% decreased markedly on day 5 in the control group and on day 3 in the DED group. Then, it gradually increased with time in both groups. On day 14 after surgery, the IOP% in the DED group was significantly higher than that in the control group. The differences in the IOP% between the two groups were not significant on days 1, 3, 5, 7, 21 and 28 after surgery. * *p* < 0.05.

The IOP percentage (IOP%) was calculated as follows: IOP% = IOP_operated_/IOP_unoperated_ [14]. The IOP% decreased markedly on day 5 in the control group and on day 3 in the DED group; then, it gradually increased over time in both groups. On day 14 after surgery, the IOP% in the DED group was significantly higher than that in the control group (*p* < 0.05). Both groups displayed higher IOP on day 28 after surgery than before surgery (Figure 5).

### 2.8. Filtering Bleb Observation

#### 2.8.1. Filtering Bleb Area

**Figure 6:**
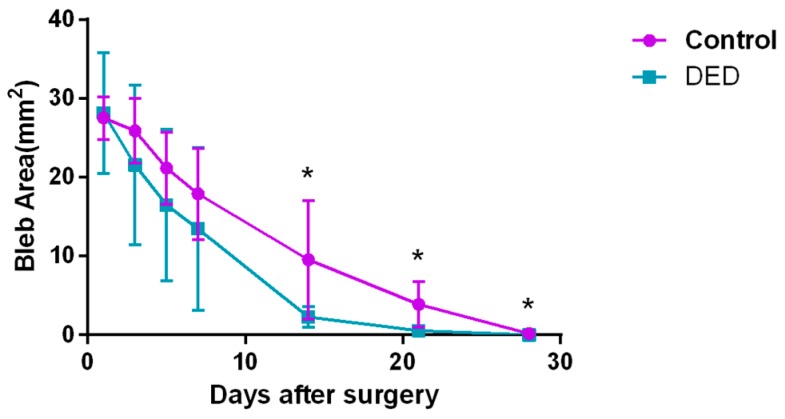
Changes in filtering bleb area over 28 days in the two groups. The filtering bleb areas shrank slowly with time in both groups. On days 14, 21 and 28 after surgery, the DED group displayed significantly smaller blebs than the control group. There were no significant differences in the bleb areas between the two groups on days 1, 3, 5 and 7 post-surgery. * *p* < 0.05.

**Figure 7:**
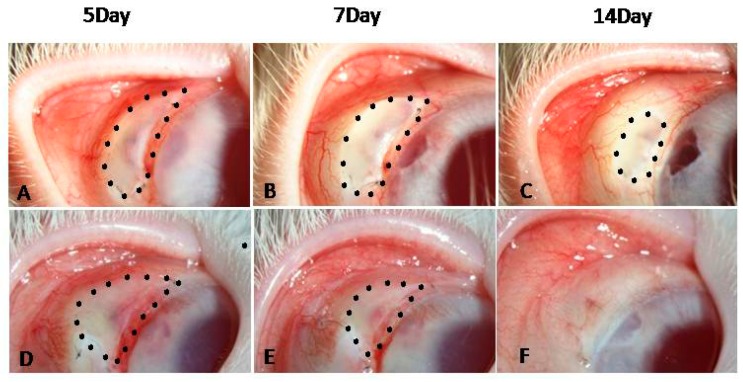
Filtering blebs on days 5, 7 and 14 after surgery. The bleb areas of the control (**A**–**C**) and DED (**D**–**F**) groups shrank gradually. On day 14, the blebs in the control group were localized, avascular, and cystic (**C**), while the blebs in the DED group were flat, scarred, and vascularized (**F**) (16×).

The filtering bleb areas of both groups shrank slowly with time. On days 14, 21 and 28 post-surgery, the DED group displayed significantly smaller blebs than the control group (*p* < 0.05, Figure 6). Figure 7 shows the post-surgical changes in bleb morphology. The control eyes displayed localized, avascular, and cystic blebs, while the DED eyes displayed flat, scarred, and vascularized blebs on day 14 post-surgery. 

#### 2.8.2. Filtering Bleb Survival Time

**Figure 8:**
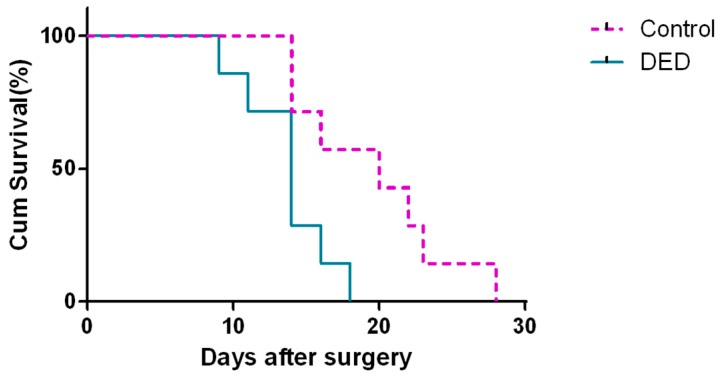
Bleb survival curves in the two groups. A Kaplan–Meier analysis showed that the survival distributions were significantly different between the control and DED groups (*p* < 0.05).

The mean bleb survival times in the control and DED groups were 19.57 ± 5.22 and 13.71 ± 2.98 days, respectively. The difference in survival time between the two groups was statistically significant (*p* < 0.05, Figure 8).

### 2.9. Histologic Evaluation of Bleb Regions

#### 2.9.1. Hematoxylin-Eosin(HE) Staining

**Figure 9:**
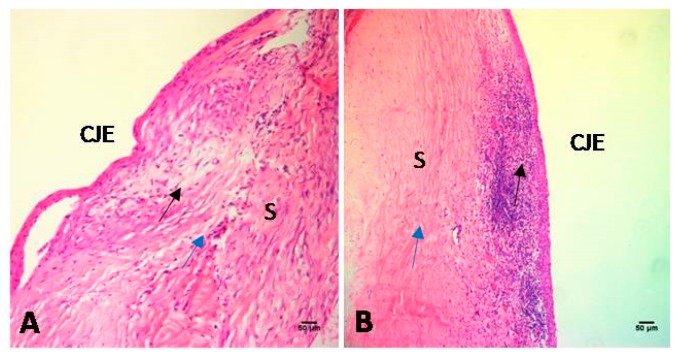
Histologic features of the filtration sites in the two groups on day 28 post-surgery. Loose subconjunctival connective tissue (black arrow), wide scleral spaces (blue arrow), and a few inflammatory cells were noted in the filtration area in the control group (**A**). However, fibroblast hyperplasia (blue arrow), narrow scleral spaces, and severe inflammatory cell infiltration (black arrow) were observed in the filtration area in the DED group (**B**) (200×). CJE: conjunctival epithelium; S: sclera. Scale bar = 50 μm.

On day 28 after surgery, HE staining revealed the presence of loose subconjunctival connective tissue, wide scleral spaces, and a few inflammatory cells in the filtration area in the control group. In contrast, HE staining revealed the presence of fibroblast hyperplasia, narrow scleral spaces, and severe inflammatory cell infiltration in the filtration area in the DED group (Figure 9).

#### 2.9.2. Immunohistochemistry for Proliferating Cell Nuclear Antigen (PCNA)

**Figure 10:**
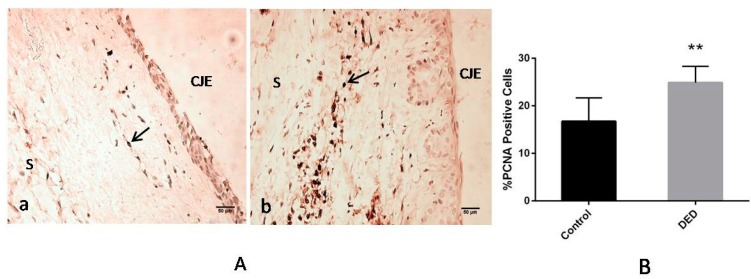
Proliferating cell nuclear antigen (PCNA) expression in the filtration blebs on day 28. (**A**) The control group (**a**) displayed reduced PCNA expression compared with that in the DED group (**b**) (400×); (**B**) the percentages of PCNA-positive cells in two groups were significantly different. Black arrows: PCNA-positive cells. CJE: conjunctival epithelium; S: sclera. ** *p* < 0.01. Scale bar = 50 μm.

The results of the immunohistochemical analysis of PCNA expression in the filtration blebs on day 28 are shown in Figure 10. A significantly greater number of cells expressing PCNA, a phenomenon indicative of greater cell division, was observed in the DED group than in the control group (*p* < 0.01, Figure 10A,B).

#### 2.9.3. Immunofluorescence for α-Smooth Muscle Actin (α-SMA)

**Figure 11:**
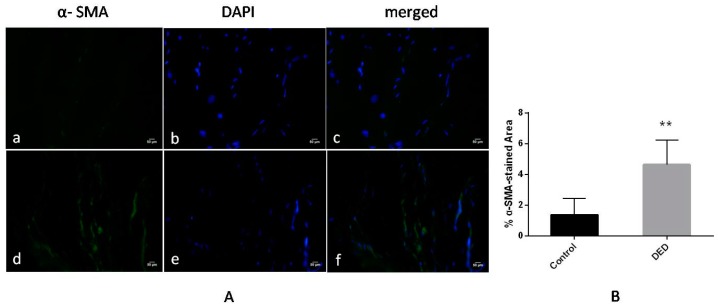
α-smooth muscle actin (α-SMA) expression in the filtration blebs on day 28 (α-SMA staining: green; nuclear staining: blue). (**A**) The α-SMA-stained area was larger in the DED group (**d**–**f**) than in the control group (**a**–**c**) (400×); (**B**) the percentage of α-SMA-stained area was significantly different between the two groups. ** *p*< 0.01. Scale bar = 50 μm.

The results of the immunofluorescence analysis of α-SMA expression in the filtration blebs on day 28 are shown in Figure 11. A significantly higher level of α-SMA-expression, which is indicative of greater fibroblast-myofibroblast transformation, was observed in the DED group than in the control group (*p* < 0.01, Figure 11A,B).

## 3. Discussion

This study shows that DED promotes filtering bleb scar formation and shortens bleb survival time; these effects may be mediated via IL-1β.

A rabbit DED model can be established via many methods, such as surgical lacrimal, meibomian and harderian gland extirpation [5,15]; lacrimal gland denervation [16]; and topical trichloroacetic acid [17,18] or BAC application [19,20]. DEWS reported that [5] the surgical removal of the lacrimal, meibomian and harderian glands could simulate the characteristics of human DED, including tear hyperosmolarity and ocular surface damage. Moreover, lacrimal gland denervation could alter tear protein and lipid profiles. However, post-surgical conjunctival scarring may interfere with filtering bleb formation and maintenance. Therefore, the two methods mentioned above were not suitable for this study. Topical administration of trichloroacetic acidor BAC is a convenient method of establishing a DED model. Long-term BAC-containing antiglaucoma eye drop treatment is an important cause of DED in patients with glaucoma [6,21,22,23]; thus, treatment with BAC was considered a better approach for establishing the rabbit DED model used in this study. Previous studies [19,24] had reported that 0.1% BAC simulated DED reasonably well by inducing chronic ocular surface injury. In the current study, eyes that were treated with twice-daily topical 0.1% BAC for 21 days displayed features characteristic of DED, including an elevated inflammatory index, CE damage, decreased basal tear secretion, goblet cell loss, and increased IL-1β levels. Chaoyang Li et al. [25] found that decreases in goblet cell density and MUC5AC levels as well as ultrastructural disorders of CE were sustained for 21 days after using the above method to establish a rabbit DED model. In our study, blebs persisted for 9 to 18 days in the DED group, which was within the valid time of this DED model.

Inflammatory cytokines, such as IL-1β, play an important role in the pathogenesis of DED [26,27]. Lam et al. [28] found that IL-1β expression levels were increased in the tears of patients with DED, and Simmons et al. [29] reported that IL-1β levels were significantly upregulated in the conjunctiva and CE of a murine dry eye model. Our study showed that rabbits with DED induced by 0.1%BAC displayed higher IL-1β levels in the CJE and CE than rabbits with normal eyes. Hyperosmolarity is a non-microbial inducer of IL-1 [30], and tear hyperosmolarity is a universally recognized characteristic of DED [5,31]. Increased tear osmolarity activates the mitogen-activated protein kinase (MAPK) signal transduction pathway, which leads to the activation of transcription factors such as activator protein-1 (AP-1) and nuclear factor kappa B (NF-κB), causing IL-1β overexpression [32,33].In local ocular surface lymph nodes, an afferent immune response mediated by IL-1β causesT helper 17 (Th17) cells to release interleukin 17A (IL-17A), leading to decreased tear film stability and ocular surface damage, thereby causing further desiccation and inflammation, which results in the formation of a positive feedback loop [34].

Chronic inflammation is a risk factor for fibrosis because it entails the release of inflammatory cytokines, such as IL-1, leading to high transforming growth factor β (TGF-β) levels [35,36]. A previous study showed that TGF-β was critically involved in conjunctival scarring after glaucoma surgery [37,38]. TGF-β induces fibroblasts and mesenchymal cells to reenter the cell cycle and transforms them into highly contractile myofibroblasts expressing α-SMA [4,39].These transformed cells cause excess extracellular matrix (ECM) accumulation by inducing ECM protein (fibronectin and collagen) hyperexpression [40]. Therefore, myofibroblasts contribute to conjunctival scar formation by enhancing tissue strength and promoting ECM accumulation. PCNA plays a vital role in fibroblast proliferation [41]. In the current study, we found that the levels of PCNA and α-SMA were significantly increased in the DED group, illustrating that fibroblast proliferation and transformation were increased in DED eyes compared with those in control eyes, resulting in shorter bleb survival times in the former group than in the latter group. Our study also showed that IL-1β levels were higher in the DED group than in the control group. Thus, we hypothesized that elevated IL-1β levels promoted TGF-β expression, as well as fibroblast proliferation and fibroblast-myofibroblast transformation—processes characterized by increased PCNA and α-SMA expression—ultimately resulting in bleb fibrosis. Currently, many studies are ongoing regarding the role of TGF-β-mediated signaling in tissue fibrosis. These studies have not examined the typical Smad pathway but have evaluated non-Smad pathways, such as the mitogen-activated protein kinase–extracellular signal-regulated kinase MAPK-ERK, p38, and c-Jun N-terminal kinase (JNK) pathways [42,43,44]. It was reported that the TGF-β/Smad3 signaling pathway played a vital role in the progression from inflammation to fibrosis [45]. However, whether increased IL-1β expression stimulates the TGF-β/Smad3 signaling pathway in the DED model, thereby causing bleb fibrosis, still requires further investigation.

## 4. Materials and Methods

### 4.1. Animals and Experimental Procedure

Sixteen New Zealand white rabbits (weight, 2.0–2.5 kg; age, 10–12 weeks) were obtained from Guangdong Medical Laboratory Animal Center, Guangzhou, China, and were maintained under standard conditions (23 ± 2 °C; relative humidity 60–10%; and 12/12-h light/dark cycles) and allowed free access to food and drink. The animals were allowed to adapt to their new environment for one week before the start of the experiments. All procedures were performed according to the ARVO Statement for the Use of Animals in Ophthalmic and Vision Research and were approved by the Committee on the Ethics of Animal Experiments of the Zhongshan Ophthalmic Center, SunYat-sen University (Permit Number: 2016-098, Guangzhou, China, 29 April 2016).

The rabbits were randomly divided into two groups (control and DED), and the left eye of each rabbit was used for the experiment. The DED model was induced by the twice-daily topical administration of 0.1% BAC drops (Sigma-Aldrich, St. Louis, MO, USA) for 3 weeks [19,25]. The rabbits in the control group did not receive any treatments. IOP measurements, inflammatory index measurements, corneal fluorescein staining, Schirmer’s test and CIC grading were performed sequentially after treatment with BAC was discontinued. One rabbit was randomly selected from each group and sacrificed, after which its cornea and conjunctiva were prepared for immunofluorescence analysis (IL-1β). Then, trabeculectomy was performed on 14 eyes from 14 rabbits. On the 1st, 3rd, 5th, 7th, 14th, 21st, and 28th days following surgery, ocular examinations assessing the indicated parameters (IOP, conjunctival congestion, flares in the anterior chamber, bleb morphology and survival time, hyphema and other phenomena) were conducted. All the rabbits were killed on the 28th postoperative day. Histologic assessments of the bleb region were performed via HE staining, immunohistochemical staining (PCNA) and immunofluorescence (α-SMA) analyses.

### 4.2. Inflammatory Index

To evaluate the severity of ocular surface inflammation after 0.1%BAC treatment, we analyzed the inflammatory index via slit-lamp examination [46]. Inflammation was scored based on the presence or absence of the following parameters: (1) ciliary hyperemia: absent, 0; present but less than 1 mm, 1; between 1 and 2 mm, 2; greater than 2 mm, 3; (2) central corneal edema: absent, 0; present and iris details are visible, 1; present and iris details are not visible, 2; present and the pupil is not visible, 3; (3) peripheral corneal edema: absent, 0; present and iris details are visible, 1; present and iris details are not visible, 2; present and the pupil is not visible, 3. The scores of the above parameters were added together and divided by 9 to obtain the final results of the experiment.

### 4.3. Fluorescein Staining of the Cornea

Two microliters of 1% fluorescein sodium (Zhongshan Ophthalmic Center, Guangzhou, China) was dropped into the conjunctival sac, after which the eye was examined using a slit lamp with a cobalt blue filter at a magnification of 16. Fluorescein staining was graded according to the following 4-tiered staining scale [47]: no staining, 0; a few punctate lesions but less than 10% coverage, 1; 10–50% coverage of the corneal surface, 2; more than 50% coverage of the corneal surface, 3.

### 4.4. Schirmer’s Tear Test

Tear volumes were measured when each rabbit was in a quiet and relaxed state. A drop of proxymetacaine hydrochloride (Alcaine 0.5%; Alcon, Fort Worth, TX, USA) was placed in each eye, and a cotton swab was used to dry any visible fluid. Then, a Schirmer tear-filter strip (Tianjin Jingming New Technological Development Co., Ltd., Tianjin, China) was inserted into the lateral lower conjunctival sac, and the wetted length was recorded after 5 min. 

### 4.5. Conjunctival Impression Cytology

After placing 0.5% Alcaine in the eye and removing any excess fluid from the eye, we gently placed acircular piece of nitrocellulose filter paper (Merck Millipore Ltd., Billerica, MA, USA) with a diameter of 3.5 mm on the superior bulbar conjunctiva. The paper was held in place for 10–15 s via the application slight pressure and was then peeled away from the eye before being immediately fixed with 95% alcohol. Periodic acid-Schiff (PAS) reagents and hematoxylin were used to stain the tissues, and goblet cell numbers were counted under a microscope (Olympus, Tokyo, Japan) at a magnification of 400. Cellular morphology was graded according to the Nelson system [13]. Three high-powered fields in each section were randomly selected, and the average number of cells was calculated for analysis.

### 4.6. Surgical Protocol

All of the rabbits were anesthetized before surgery with an intramuscular injection of Sumianxin (0.1–0.2 mL/kg) and an intravenous injection of 3% pentobarbital (30 mg/kg). In addition, 0.5%Alcaine eye drops were used as a local anesthetic. Glaucoma filtration surgery was performed via the following procedure: (1) a 6-mm fornix-based conjunctival flap was produced in the superior nasal quadrant of the left eye; (2) a 1/2-thickness triangular scleral flap was created; (3) a small piece of lint soaked in 0.25 mg/mL mitomycin C was placed over the scleral bed for 3 min; (4) a 2 mm × 1 mm piece of tissue containing the deep sclera, trabeculum, and peripheral cornea was excised, and a peripheral iridectomy was performed; and (5) a 10–0 nylon suture was used to close the conjunctival incision to ensure water-tight closure. Tobramycin eyedrops (2 times daily) and tobramycin ointment (once daily) were applied to the eye for 1 week after surgery. All operations were performed by an experienced ophthalmologist. 

### 4.7. Clinical Evaluation of the Effects of Surgery

A non-invasive rebound tonometer (Icare, Espoo, Finland) was used to measure IOP. After the rabbit’s head was fixed, the tip of the probe was placed perpendicularly on the surface of the central cornea, after which the results of the test were recorded. Each eye was measured 3 times, and the average of the three measurements was used in the analysis. A slit-lamp examination was performed to determine whether anterior chamber inflammation was present and to assess bleb morphology and determine survival time. Flat, vascularized, and scarred blebs were considered failed blebs [48]. The bleb length and width were measured with a Verniercaliper, and the bleb area was calculated according to the method reported by Cordeiro et al. [49].

### 4.8. Histologic Evaluation

The rabbits were euthanized humanely, after which the left eyes were enucleated and immersed in 4% paraformaldehyde at 4 °C. The tissues within the bleb region were resected and embedded in paraffin. After microtome sectioning, the samples were stained with HE to gain a general impression of the total cellularity of each sample. IL-1β (cornea and conjunctiva) and α-SMA (bleb region) were detected by immunofluorescence staining. The frozen sections were dried at room temperature (RT), washed three times with phosphate-buffered saline (PBS), blocked with goat serum for 1 h and then incubated with anti-IL-1β (1:200, Abcam, Cambridge, UK) and anti-α-SMA (1:100, Abcam, Cambridge, UK) antibodies for 14 to 18 h at 4 °C. After 3 more washes in PBS, the samples were incubated with Alexa 488 goat anti-rabbit/mouse IgG secondary antibodies (1:1000, Invitrogen, Carlsbad, CA, USA) for 1 h at RT before undergoing an additional 3 washes in PBS. Then, 4′,6-diamidino-2-phenylindole dihydrochloride (DAPI) was used to stain the nuclei (1:1000, Sigma-Aldrich, St. Louis, MO, USA). The percentages of α-SMA-stained areas in five high-powered fields in each section were quantified using Image J software (1.50i, National Institutes of Health, Bethesda, MD, USA). PCNA expression was measured by immunohistochemistry to determine whether recent cell division had occurred at the site of the surgical wound. After deparaffinization, the tissue sections were placed in 0.01% mol/L citrate buffer and then heated to boiling in a microwave oven. The sections were then allowed to cool at RT, and 3% H_2_O_2_ was used to inactivate endogenous peroxidases. The tissues were subsequently blocked with 5% BSA and incubated with anti-PCNA primary antibody (prediluted, Abcam, Cambridge, UK) for 14 to 18 h at 4 °C. After being washed with PBS, the sections were incubated with the corresponding secondary antibodies for 1 h. The reaction product was developed with diaminobenzidine. The percentages of PCNA-positive cells in five high-powered fields in each section were calculated using Image-Pro Plus software (6.0, Media Cybernetics, Silver Spring, MD, USA).

### 4.9. Statistical Analysis

A statistical analysis was performed to determine the differences in the inflammatory index, fluorescein staining score, Schirmer’s tear test results, goblet cell counts, IOP%, bleb areas and survival times, and the percentages of PCNA-positive cells and α-SMA-stained areas between the two groups (SPSS 24.0 software; SPSS Inc., Chicago, IL, USA). A Student’s *t*-test was performed for two-group comparisons. Differences in bleb survival times were tested using a Kaplan–Meier analysis. *p* < 0.05 was considered statistically significant.

## 5. Conclusions

In summary, our study evaluated the effect of DED on bleb scar formation in rabbit glaucoma filtration surgery. We found that DED promoted scar formation of filtering blebs and shortened bleb survival time, effects that may be mediated via IL-1β. Therefore, we believe that DED screening and treatment may improve glaucoma filtering surgery outcomes and may provide clinicians with useful information for the optimization of operative treatments in patients with glaucoma.

## Abbreviations

DED	Dryeye disease
BAC	Benzalkonium chloride
IL-1β	Interleukin-1β
PCNA	Proliferating cell nuclear antigen
α-SMA	α-smooth muscle actin
PAS	Periodic acid-Schiff
DEWS	Dry Eye Work Shop
IOP	Intraocular pressure
NSAID	Nonsteroidal anti-inflammatory drug
CIC	Conjunctivalimpression cytology
CJE	Conjunctival epithelium
CE	Corneal epithelium
HE	Hematoxylin-Eosin
MAPK	Mitogen-activated protein kinase
CIC	Conjunctivalimpression cytology
AP-1	Activator protein-1
NF-κB	Nuclear factor κ B
Th17	Thelper 17 cell
IL-17A	Interleukin-17A
TGF-β	Transforming growth factor β
ECM	Extracellular matrix
MAPK-ERK	Mitogen-activated protein kinase–extracellular signal-regulated kinase
JNK	c-Jun N-terminal kinase
RT	Room temperature
PBS	Phosphate-buffered saline
DAPI	4′,6-diamidino-2-phenylindole dihydrochloride
